# Switching from biosimilar (Basalin) to originator (Lantus) insulin glargine is effective in Chinese patients with diabetes mellitus: a retrospective chart review

**DOI:** 10.12688/f1000research.13923.1

**Published:** 2018-04-18

**Authors:** Xia Hu, Lei Zhang, Yanhu Dong, Chao Dong, Jikang Jiang, Weiguo Gao

**Affiliations:** 1 Qingdao Endocrine and Diabetes Hospital, Qingdao, 266071, China

**Keywords:** Insulin glargine, biosimilar, diabetes mellitus, Lantus, Basalin

## Abstract

**Background: **This study investigated the effectiveness and safety of switching from Basalin® to Lantus® in Chinese patients with diabetes mellitus (DM).

**Methods: ** A retrospective chart review conducted using the electronic medical records of patients hospitalized at the Qingdao Endocrine and Diabetes Hospital from 2005 to 2016. All patients were diagnosed with DM and underwent switching of insulin from Basalin to Lantus during hospitalization. Data collected included fasting (FBG), pre- and post-prandial whole blood glucose, insulin dose, reasons for insulin switching and hypoglycemia. Four study time points were defined as: hospital admission, Basalin initiation, insulin switching (date of final dose of Basalin), and hospital discharge. Blood glucose measurements were imputed as the values recorded closest to the dates of these four time points for each patient.

**Results: **Data from 73 patients (70 patients with type 2 diabetes, 2 with type 1, and 1 undisclosed) were analyzed. At admission, mean glycated hemoglobin (HbA1c) and FBG were 8.9% (SD=1.75) and 9.98 (3.22) mmol/L, respectively. Between Basalin initiation and insulin switch, mean FBG decreased from 9.68 mmol/L to 8.03 mmol/L (p<0.0001), over a mean 10.8 (SD=6.85) days of Basalin treatment, and reduced further to 7.30 mmol/L at discharge (p=0.0116) following a mean 6.6 (7.36) days of Lantus. The final doses of Basalin and Lantus were similar (0.23 vs. 0.24 IU/kg/day; p=0.2409). Furthermore, reductions in pre- and post-prandial blood glucose were also observed between Basalin initiation, insulin switch and hospital discharge. The incidence of confirmed hypoglycemia was low during Basalin (2 [2.4%]) and Lantus (1 [1.2%]) treatment, with no cases of severe hypoglycemia.

**Conclusion: **In this study population, switching from Basalin to Lantus was associated with further reductions in blood glucose, although the dose of insulin glargine did not increase. Further studies are required to verify these findings and determine the reason for this phenomenon.

## Introduction

In China, insulin is more widely used to treat patients with type 2 diabetes mellitus (T2DM) compared with many Western countries ^1, 2^. Chinese patients with T2DM are characterized by a relatively young age of diabetes onset, low bodyweight, and early β-cell dysfunction combined with insulin resistance ^2^. Furthermore, progressive deterioration of β-cell function is known to contribute to the increasing difficulty of achieving glycemic control experienced during the T2DM disease course ^3– 5^. Therefore, interventions to preserve β-cells are recognized to be effective and, considering the early β-cell dysfunction which characterizes Chinese T2DM patients, can be especially effective in this population ^6^. However, challenges for implementing insulin treatment in China include lack of patient education, limitations of medical resources, inadequate health care systems making patient follow up difficult, and suboptimal blood glucose monitoring ^1^. Therefore, Chinese physicians often prefer to initiate intensified treatment regimens in an inpatient setting to allow close control of treatment, and monitoring of effectiveness and safety. Given this situation, early initiation of insulin treatment in an inpatient setting is often favored in China for the treatment of patients with T2DM. Several studies in Chinese patients with newly-diagnosed T2DM have shown that early intensive insulin treatment preserves β-cell function and leads to glycemic remission ^7– 9^. Furthermore, a meta-analysis of previous studies showed that 46.3% of Chinese patients with T2DM who received intensive insulin treatment achieved glycemic remission after 12 months, without the use of anti-diabetic medication and relying solely on lifestyle modification to control blood glucose levels ^10^.

Basal insulin is recommended as a convenient way to initiate insulin treatment by most international treatment guidelines, and by the Chinese Diabetes Association ^11– 13^. ( see the International Diabetes Federations Diabetes Atlas, 7 ^th^ edition) As reported by the large, observational ORBIT study, insulin glargine is the most popular basal insulin for the initiation of insulin treatment in Chinese patients with T2DM (71%), followed by detemir (13%) and neutral protamine Hagedorn (NPH)(16%) insulin. In China, there are two available versions of insulin glargine; the originator Lantus® (Sanofi), and the biosimilar Basalin® ( see Gan & Lee Pharmaceuticals history page), which has been commercially available since 2005 in China, Egypt, Pakistan, South East Asia, and countries in Latin America ^14^. Basalin was the first biosimilar of insulin glargine marketed in China, and has since become a commonly used treatment option for many Chinese physicians. However, while biosimilars are developed to be highly similar to originator therapies, due to the degree of natural variability inherent to biological drugs, which are produced using living organisms, and because of differences in manufacturing processes between products, they are not identical to the original therapy ^15^. Results from a previous head-to-head study of Basalin and Lantus in Chinese people with T2DM showed equivalent glycemic control and safety, and a further crossover study in Chinese people with T2DM using a continuous glucose monitoring system (CGMS) reported non-inferiority of Basalin to Lantus for mean blood glucose, blood glucose fluctuations and rates of hypoglycemia ^16, 17^. However, other than these two previous reports, there are no further confirmatory studies in real-world clinical practice. In addition, a local-language case report described improved blood glucose control and reduced insulin dose in three Chinese patients with T2DM who switched from Basalin to Lantus due to suboptimal glycemic control ^18^. Given this situation, it is important to further verify the findings of this case report in a larger population of patients who switched from Basalin to Lantus in a real-life clinical practice setting. We therefore hypothesized that patients with diabetes who switch from Basalin to Lantus in a real-world setting may achieve improved control of blood glucose with a similar safety profile.

## Methods

### Study design and patients

This was a retrospective chart review conducted based on the electronic medical records (EMRs) of patients hospitalized at the Qingdao Endocrine and Diabetes Hospital from 2005 to 2016. Qingdao Endocrine and Diabetes Hospital is a public, non-profit, tertiary hospital that was established in 2002 and is located in Qingdao city, one of the biggest cities on the east coast of China. The Hospital is the only specialist diabetes hospital, not only in Qingdao, but also throughout the region, with a catchment area of approximately 100 kilometers, which covers 7.2 million people. The majority of the patients treated in the Hospital are Han Chinese residing in Qingdao city and the surrounding regions. Less than 1% of patients are national ethnic minorities or of foreign origin. In general, patients are hospitalized due to suspected acute diabetic complications including ketosis, ketoacidosis, diabetic hyperosmolality, uncontrolled hyperglycemia and at least one chronic complication, or significant worsening of chronic complications of diabetes (including rental hemorrhage, significant elevated albuminuria and/or serum creatinine, acute myocardial infarction, etc.).

**Figure 1.**
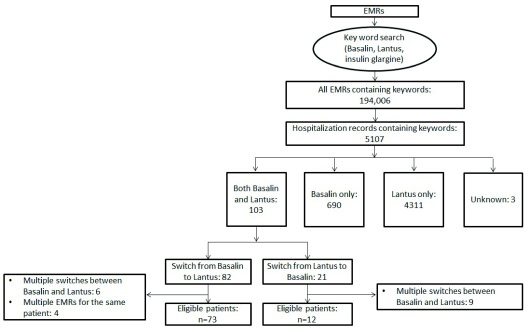
Data selection flow diagram.

The data selection process is summarized in Figure 1. EMRs were first searched using the keywords “Lantus”, “Basalin” or “insulin glargine” to identify EMRs with recorded use of insulin glargine. EMRs were then subdivided based on the type of insulin glargine used; Lantus and Basalin (103), Basalin only (690), Lantus only (4311), or no information about which type of insulin glargine had been used (3). The EMRs with recorded use of both Lantus and Basalin were further sorted into two groups; switch from Basalin to Lantus (82) and switch from Lantus to Basalin (21). Finally, the EMRs were evaluated by hand to exclude cases involving multiple switches between Basalin and Lantus, and identify multiple EMRs for the same patient on different hospitalizations (in such instances only the case from the most recent hospitalization was included in the analysis). Of the 21 EMRs including a switch from Lantus to Basalin, nine reported multiple switches of insulin, and therefore only 12 EMRs (each representing one patient) were eligible for inclusion, and these data were not included in the present analysis. Of the 82 cases including switching from Basalin to Lantus, six records included multiple switches of insulin, and one subject had been hospitalized four times, and therefore had four medical records; therefore, a total of 73 patients who switched from Basalin to Lantus were included.

**Figure 2.**
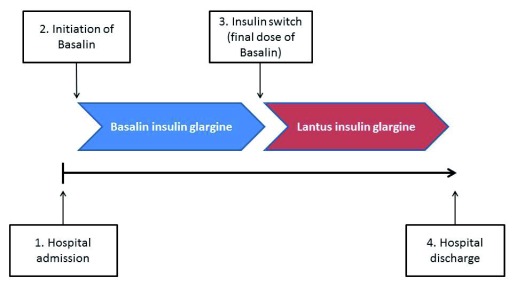
Study schematic showing the four study time points.

The key objective of the present analysis was to evaluate the change in FBG for patients with DM after switching from Basalin to Lantus. Secondary aims of the analysis included evaluation of changes in seven-point blood glucose, basal and prandial insulin dose, and hypoglycemia after switching from Basalin to Lantus. Four study time points were defined as hospital admission, Basalin initiation, insulin switching (the date of the final dose of Basalin), and hospital discharge ( Figure 2). For each study subject, values of blood glucose at hospital admission and hospital discharge were imputed as the measurements recorded closest to the date of hospital admission and discharge, and values at Basalin initiation and insulin switch were defined as the measurements recorded closest to the date of the first and last doses of Basalin, respectively. The duration of insulin glargine treatment was calculated as the end date of the final dose minus the start date of the initial dose +1 day. If the date of the final dose of Lantus was not recorded then the final dose date was taken as the date of hospital discharge.

The study protocol was approved by the Ethical Review Board of Qingdao Endocrine and Diabetes Hospital in a regular meeting during October 2016 (Document No 2016-10-1). All procedures followed were in accordance with the ethical standards of the responsible committee on human experimentation (institutional and national) and with the Helsinki Declaration of 1964, as revised in 2013. As this was a retrospective observational study based on electronic medical records it was not possible to contact each patient for individual consent, however the researchers received anonymized data from the hospital's IT department with permission from both the Ethical Review Board and the Principal of the Hospital.

### Study treatments

Basalin (Gan & Lee Pharmaceutical, Beijing, China) and Lantus (Sanofi, Paris, France) are both provided in a 100 IU/mL concentration by the manufacturers. Both insulin glargines are provided with an injection pen; the GanLee Pen (Gan & Lee Pharmaceutical) for Basalin and the SoloSTAR®/ClikSTAR® (Sanofi) for Lantus.

### Measurements and data collection

Data were retrospectively harvested from EMRs collected in the hospital information system and included demographics, medical history, details of medication including pre-hospitalization treatment regimen and concomitant use of oral antidiabetic drugs (OAD), laboratory measurements such as glycated hemoglobin (HbA1c) and daily blood glucose measurements, and safety data. Raw data were encoded by a trained member of staff. Daily blood glucose control was evaluated using seven-point blood glucose, comprised of fasting blood glucose levels (FBG) before breakfast, and blood glucose levels post-breakfast, pre- and post- lunch and dinner, and pre-bed.

Blood glucose measurements were conducted by hospital staff from finger-stick blood samples and using the Nova StatStrip system (Nova Biomedical, MA, USA). Hypoglycemia was defined as any recorded incidence of blood glucose ≤3.9 mmol/L, and severe hypoglycemia as blood glucose ≤2.8 mmol/L.

### Statistical methods

Two subgroup analyses were performed; in patients who received basal-bolus therapy with Basalin and prandial insulin as their initial therapy (excluding patients who received OADs), and for patients who switched from Basalin to Lantus due to suboptimal glycemic control as recorded in their medical record.

Data were summarized using descriptive statistics. Continuous variables are presented as mean (standard deviation [SD]), and discrete variables are summarized as frequency and percentage. Paired Student’s t-tests were used to evaluate differences between variables. A p-value <0.05 was considered statistically significant. All analyses were conducted using SAS software (version 9.4).

## Results

### Patients

At baseline, the majority of the study patients were middle aged or older, with a mean body mass index of 27.4 kg/m ^2^ and a mean duration of diabetes mellitus (DM) of 9.1 years ( Table 1). Metabolic control was poor in most patients, indicated by an average HbA1c of 8.9%, and FBG of 9.98 mmol/L, at hospital admission. Diabetic complications had been reported in 86.3% of patients, and 38.4% had a history of cardiovascular disease. The mean duration of hospitalization was 22.3 days. Initial treatment with Basalin insulin glargine was received in combination with prandial insulin for the majority of patients (84.9%). The most common reason for switching to Lantus as recorded in patients’ medical records was suboptimal glycemic control (30.1%), followed by patient decision (4.1%).

**Table 1.** Clinical characteristics of patients who underwent switching from Basalin to Lantus insulin glargine.

Variable ^a^	N=73
Classification of diabetes mellitus, n (%)	
Type 1	2 (4)
Type 2	70 (96)
Unclassified	1
Age, years	59.8 (13.10)
Female, n (%)	39 (53.4)
Height, cm	164.5 (8.55)
Body mass index, kg/m ^2^	27.4 (3.57)
Fasting blood glucose, mmol/L	9.98 (3.22)
Duration of diabetes mellitus, years	9.1 (5.92)
Hemoglobin A1c at hospital admission, %	8.9 (1.75)
Diabetic complications ^b^, n (%)	63 (86.3)
History of cardiovascular disease, n (%)	28 (38.4)
Hypertension, n (%)	52 (71.2)
History of smoking, n (%)	14 (19.2)
Medication regimen before hospitalization, n (%)	
OADs	30 (41.1)
Premixed insulin	25 (34.3)
Insulin glargine + OADs	5 (6.9)
Basalin insulin glargine + OADs	3 (4.1)
Basal + prandial insulin	1 (1.4)
Other or data not available	9 (12.3)
Days of hospitalization, days ^c^	22.3 (12.02)
Initial treatment regimen during hospitalization, n (%)	
Basalin insulin glargine + prandial insulin	62 (84.9)
Basalin insulin glargine + OADs	11 (15.1)

^a^Data are summarized as mean (SD) unless stated; ^b^Any diabetic complication, yes or no; ^c^patients may have received other treatment before initiating biosimilar insulin glargine. OADs, oral antidiabetic drugs.

### Glycemic control

Overall, mean FBG decreased from initiation of Basalin insulin glargine until hospital discharge ( Table 2). From initiation of treatment with Basalin insulin glargine until insulin switch, FBG decreased by 1.65 mmol/L; from 9.68 mmol/L to 8.03 mmol/L (p<0.0001). From insulin switch until hospital discharge (while patients were receiving Lantus), FBG reduced by a further 0.73 mmol/L, to 7.30 mmol/L (p=0.0116).

**Table 2.** Seven-point blood glucose summary.

	Study time point (n=73)				Inter-group comparison p-values ^b^		
Blood glucose, mmol/L ^a^	Hospital admission	Basalin initiation	Insulin switch	Hospital discharge	Basalin initiation vs. hospital admission	Insulin switch vs. Basalin initiation	Hospital discharge vs. insulin switch
FBG	9.98 (3.22)	9.68 (3.10)	8.03 (2.39)	7.30 (1.62)	0.1650	<0.0001	0.0116
Post-breakfast BG	12.32 (3.13)	11.95 (3.27)	10.59 (3.28)	9.62 (2.40)	0.1644	0.0126	0.0164
Pre-lunch BG	10.96 (4.07)	10.25 (3.56)	8.70 (2.85)	8.13 (2.82)	0.1050	0.0003	0.0312
Post-lunch BG	12.09 (4.14)	11.33 (4.29)	9.80 (2.65)	9.49 (2.55)	0.0270	0.0024	0.3277
Pre-dinner BG	11.07 (3.71)	10.50 (3.75)	8.82 (2.96)	8.38 (2.55)	0.0578	0.0005	0.1054
Post-dinner BG	12.41 (4.00)	11.80 (3.64)	9.92 (2.53)	9.27 (2.22)	0.0492	<0.0001	0.0291
Pre-bedtime BG	10.50 (3.57)	10.38 (3.52)	8.65 (2.80)	7.90 (1.92)	0.6705	<0.0001	0.0088

^a^Data are summarized as mean (SD) unless stated; ^b^calculated using a two-tailed Student’s t-test. BG, blood glucose; FBG, fasting blood glucose.

Mean post-prandial blood glucose values indicated improved glycemic control during treatment with Basalin, and further improvements after switching to Lantus ( Table 2, Supplementary Figure 1). Seven-point blood glucose decreased at all four study time points from hospital admission to hospital discharge. Between Basalin initiation and insulin switch the reductions in mean seven-point blood glucose were significant across all seven blood glucose measurements (p<0.05). Subsequently, between insulin switch and hospital discharge, reductions in mean seven-point blood glucose values were significant (p<0.05) at all times except post-lunch and pre-dinner.

### Insulin dose during hospitalization

Subjects received Basalin insulin glargine for a mean duration of 10.8 (6.85) days, and during this time the mean insulin dose increased significantly from 0.19 IU/kg/day to 0.23 IU/kg/day (p<0.0001)( Table 3). Lantus insulin glargine was received for a mean duration of 6.6 (7.36) days; the initial mean dose was 0.24 IU/kg/day and the final dose before hospital discharge was 0.24 IU/kg/day (p=0.8720). In addition, the mean final doses of Basalin and Lantus were similar (p=0.2409).

**Table 3.** Insulin doses during initial treatment with Basalin insulin glargine and after switching to Lantus.

Insulin dose, IU/kg/day	Basalin insulin glargine (mean duration 10.8 [6.85] days)		Insulin switch	Lantus insulin glargine (mean duration 6.6 [7.36] days)		P-value ^a^ (Basalin final dose vs. originator final dose)
	Initial dose	Final dose		Initial dose	Final dose	
Insulin glargine	0.19 (0.07)	0.23 (0.08)		0.24 (0.08)	0.24 (0.09)	0.2409
P-value, initial vs. final dose ^a^		<0.0001			0.8720	
Prandial insulin ^b^	0.32 (0.08)	0.40 (0.15)		0.41 (0.15)	0.40 (0.16)	0.8429
P-value, initial vs. final dose ^a^		<0.0001			0.4830	

^a^Paired Student’s t-test; ^b^for patients receiving basal + prandial regimens.

For patients receiving prandial insulin, the mean dose of prandial insulin increased from 0.32 IU/kg/day to 0.40 IU/kg/day between the initial and final dose of Basalin (p<0.0001), and decreased from 0.41 IU/kg/day to 0.40 IU/kg/day between initial and final dose of Lantus (p<0.4830).

### Subgroup analyses

The 62 patients who received basal-bolus (basal insulin plus multiple daily injections of prandial insulin) therapy experienced significant reductions in FBG from initiation of Basalin to hospital discharge. Between initiation of Basalin therapy and insulin switch mean FBG decreased from 10.03 mmol/L to 8.25 mmol/L (p=0.0001), and from insulin switch to hospital discharge decreased further to 7.49 mmol/L (p=0.0217) ( Table 4, Supplementary Figure 2). In this subgroup, overall glycemic control was also improved during treatment with Basalin, and continued to improve after switching to Lantus. Between initiation of Basalin and insulin switch, seven-point blood glucose reduced significantly at all seven measurements, and from insulin switch to hospital discharge decreased significantly at all measurements except post-lunch and pre- and post-dinner. The final mean doses of Basalin and Lantus were similar (0.24 [0.08] IU/kg/day vs. 0.25 [0.09] IU/kg/day; p=0.1321), and the final mean dose of prandial insulin at the last administrations of Basalin (0.4 [0.15]) and Lantus (0.4 [0.16]) were also similar (p=0.8429) ( Supplementary Table 1).

**Table 4.** Seven-point blood glucose for patients who received basal-bolus treatment only with no OAD use.

	Study time point (n=62)				Inter-group comparison p-values ^b^		
Blood glucose, mmol/L ^a^	Hospital admission	Basalin initiation	Insulin switch	Hospital discharge	Basalin initiation vs. hospital admission	Insulin switch vs. Basalin initiation	Hospital discharge vs. insulin switch
FBG	10.29 (3.33)	10.03 (3.17)	8.25 (2.51)	7.49 (1.65)	0.3008	0.0001	0.0217
Post-breakfast BG	12.51 (3.15)	12.14 (3.37)	10.87 (3.39)	9.71 (2.44)	0.2238	0.0406	0.0129
Pre-lunch BG	11.37 (4.17)	10.69 (3.54)	8.9 (2.85)	8.21 (2.88)	0.1663	0.0002	0.0207
Post-lunch BG	12.54 (4.24)	11.77 (4.43)	9.96 (2.72)	9.46 (2.62)	0.0389	0.0021	0.1292
Pre-dinner BG	11.54 (3.71)	11.04 (3.73)	9.12 (3.05)	8.6 (2.65)	0.1248	0.0005	0.0993
Post-dinner BG	12.97 (4.01)	12.27 (3.64)	9.91 (2.6)	9.28 (2.19)	0.0514	<.0001	0.0542
Pre-bedtime BG	10.89 (3.65)	10.71 (3.64)	8.77 (2.93)	7.95 (1.89)	0.6017	<.0001	0.0133

Among the 22 patients who switched to Lantus due to suboptimal glycemic control as recorded in their medical records, FBG decreased from 10.17 (3.59) mmol/L to 8.51 (2.18) mmol/L (p=0.0540) during treatment with Basalin, and between insulin switch and hospital discharge decreased further to 7.37 (1.70) mmol/L (p=0.0337) ( Supplementary Table 2). Among these patients, an overall trend towards reduction in seven-point blood glucose was also observed. Similar to the results in the main analysis population, the mean final doses of Basalin and Lantus were comparable (0.25 [0.08] IU/kg/day vs. 0.26[0.1] IU/kg/day; p=0.5069), as were the mean final doses of prandial insulin at final administrations of Basalin and Lantus (0.43 (0.13) IU/kg/day vs. 0.41 [0.14] IU/kg/day; p=0.5085) ( Supplementary Table 3).

### Safety

Hypoglycemia was reported by 2 (2.4%) of patients during treatment with Basalin insulin glargine, and by 1 (1.2%) of patients during treatment with Lantus. No patients experienced severe hypoglycemia during hospitalization.

Switching from biosimilar (Basalin) to originator (Lantus) insulin glargine, propensity score matching with readme file explain the analysis Click here for additional data file. Copyright: © 2018 Hu X et al. 2018 Data associated with the article are available under the terms of the Creative Commons Zero "No rights reserved" data waiver (CC0 1.0 Public domain dedication).

## Discussion

Our retrospective study found that Chinese patients with T2DM who initiated Basalin with pre-prandial insulin or OADs achieved further reductions in overall hyperglycemia, especially fasting hyperglycemia, after switching to Lantus (8.03 mmol/L vs. 7.30 mmol/L; p=0.0116) with a similar mean basal insulin dose (0.23 IU/kg/day vs. 0.24 IU/kg/day; p=0.8720). This result is consistent with a previous case series that found three Chinese patients with T2DM inadequately controlled with Basalin achieved improvements in glycemic control after switching to Lantus, with a similar dosage of insulin ^18, 19^. Patients in our study were relatively young (59.8 years) with a moderate disease course (9.1 years), poor glycemic control (HbA1c of 8.9%) and a high proportion of existing diabetic complications (86.3%) but a comparatively low prevalence of cardiovascular disease (38.4%). This may explain why the clinical decision was made to switch to Lantus, after achieving sub-optimal glycemic control with Basalin.

In contrast to our findings, a recently published blinded crossover study showed non-inferiority in terms of mean blood glucose, glucose fluctuations and rates of hypoglycemia as measured by CGMS for Chinese outpatients with T2DM receiving Basalin or Lantus ^16^. Although patients in this previous study had a similar mean age, BMI and duration of T2DM as our study, there were several important differences. Firstly, patients in this previous study had a relatively high mean blood glucose level during the study period in both the Basalin group and Lantus group (9.35 mmol/L vs. 9.67 mmol/L, respectively; p=0.387), especially a high FBG concentration of nearly 10 mmol/L. Secondly, no insulin titration scheme was used and it was not a treat-to-target study, in contrast to our study which utilized insulin dose titration. Finally, the duration of the previous study was 5 days with crossover on day 3, and 2 days of insulin administration before and after cross over, which may not have allowed adequate time for a full washout of the initial insulin type.

Patients in this study had poor glycemic control at Baseline (HbA1c=8.9%) and required intensified therapy to rapidly lower blood glucose levels, despite the majority having already received treatment for T2DM. Previous studies in hospitalized Chinese patients with poorly-controlled T2DM receiving intensive insulin therapy have shown that glycemic control targets based on blood glucose levels (FBG and post-prandial glucose [PPG]) can be reached in around 4 days ^20, 21^. In comparison, the mean duration of Basalin (10.8 days) and Lantus (6.6 days) insulin glargine in the present study was sufficient to allow glycemic control targets to be reached. Additionally, it should be noted that given the short-term nature of the intensified insulin treatment being assessed in this study, the use of FBG as the primary assessment of glycemic control is justified versus other longer-term biomarkers such as HbA1c, and a precedent has been set for this in previous studies of short-term intensive insulin treatment ^7– 9^.

Evidence suggests that intensive insulin treatment can protect and improve β-cell function through achievement of optimal glycemic control ^10, 22, 23^. Furthermore, initiation of a basal-bolus insulin regimen is recommended by most treatment guidelines for treatment intensification in patients with T2DM who cannot achieve glycemic control with OADs and basal insulin and is also recommended for hospitalized patients ^11– 13, 24^. The majority of patients in this study initiated treatment with a basal-bolus insulin regimen and achieved reductions in mean seven-point blood glucose during Basalin treatment, and further reductions following the switch to Lantus with a similar final dose of prandial insulin to that recorded at the end of Basalin treatment (0.40 IU/kg/day at both time points). This suggests that the further reductions in FBG achieved with Lantus treatment contributed to the overall control of hyperglycemia. As the glycemic profile of diabetes patients consists of basal glycemia, basal hyperglycemia and postprandial hyperglycemia, reductions of “basal” hyperglycemia which is often expressed as “fasting” blood glucose levels have been hypothesized to contribute to a reduction of post-prandial hyperglycemic excursions ^25^. In addition, despite the use of an intensive basal-bolus regimen in the majority of patients, the incidence of confirmed hypoglycemia was similarly low during treatment with Basalin and Lantus (2.4% vs. 1.2%).

To determine the potential confounding effects of concomitant OAD and prandial insulin use on glycemic control associated with basal insulin , we conducted two subgroup analyses in patients treated with a purely basal-bolus regimen with no OAD use and patients with recorded switching from Basalin to Lantus due to suboptimal glycemic control, and in both of these subgroups mean FBG was lower after switching from Basalin; 8.25 mmol/L vs. 7.49 mmol/L (p=0.0217) and 8.51 mmol/Lvs.7.37 mmol/L (p=0.0337). Furthermore, the results of the Joint Asia Diabetes Evaluation (JADE) study also reported that biosimilar insulin use in Asia is associated with suboptimal glycemic control, indicated by higher FBG and HbA1c levels and a lower proportion of patients achieving glycemic control targets (HbA1c <7.0%) compared with originator insulin (13.6% vs. 17.2%) ^19^.

There are several limitations of this analysis which deserve discussion. Firstly, this was a retrospective study that had many potential confounding factors including patient age, duration of DM, diabetic complications and co-morbidities, which may have affected treatment outcomes, insulin dose and safety. However, patients acted as their own controls, which reduces the effect of these confounding factors somewhat, particularly because most of the confounding factors would be constant over the relatively short mean durations of treatment and hospital stay. Secondly, all patients in this study were hospitalized with strictly-controlled diet, exercise and monitoring of blood glucose, which would be expected to have a positive effect on glycemic control independently of basal insulin treatment, and it may also be hypothesized that switching to a different basal insulin perceived as ‘superior’ could lead to a placebo effect. Although these are potential confounding factors for attributing improvements in glycemic control to basal insulin treatment alone, it should be noted that patients achieved sub-optimal glycemic control with Basalin during the initial part of their hospital stay (an average of 10 days) during which modified diet and lifestyle would be expected to have the largest positive impact on blood glucose levels. Finally, although this was a single-center study with limited data the patient profile is similar to that reported in the Fine Asia and ORBIT studies, which indicated our findings potentially have treatment implications generalizable to the broader population of Chinese patients with T2DM ^26, 27^.

In conclusion, this study provides evidence from real-world clinical practice that switching from Basalin to Lantus is associated with an improvement in blood glucose levels, with a similar insulin dose, in hospitalized patients with T2DM. Despite the limitations of the present analysis, these findings warrant further investigation by crossover randomized controlled study or further real-world evidence from a larger population.

## Data availability

The data referenced by this article are under copyright with the following copyright statement: Copyright: © 2018 Hu X et al.

Data associated with the article are available under the terms of the Creative Commons Zero "No rights reserved" data waiver (CC0 1.0 Public domain dedication). 

Dataset 1: Switching from biosimilar (Basalin) to originator (Lantus) insulin glargine, propensity score matching with readme file explain the analysis 10.5256/f1000research.13923.d199674 ^28^

## Supplementary material

**Supplementary File 1 – Document containing the following Tables and Figures:**

**Supplementary Table 1.** Summary of insulin doses during initial treatment with Basalin insulin glargine and after switching to Lantus for patients who received basal-bolus therapy only without OADs

**Supplementary Table 2.** Seven-point blood glucose summary for patients who switched due to suboptimal glycemic control as recorded in medical records

**Supplementary Table 3.** Summary of insulin doses during initial treatment with Basalin insulin glargine and after switching to Lantus for patients who switched to Lantus due to suboptimal glycemic control as recorded in medical records

**Supplementary Figure 1.** Seven-point blood glucose measurement for Chinese patients with diabetes mellitus at hospital admission, Basalin insulin glargine initiation, insulin switch, and hospital discharge

**Supplementary Figure 2.** Seven-point blood glucose measurement for Chinese patients with diabetes mellitus who received basal-bolus treatment only with no OAD use (n=62) at hospital admission, Basalin insulin glargine initiation, insulin switch, and hospital discharge
