# Supplementary material for: Switching from biosimilar (Basalin) to originator (Lantus) insulin glargine is effective in Chinese patients with diabetes mellitus: a retrospective chart review
Source: F1000Res. 2018 Apr 18;7:477. [Version 1] doi: 10.12688/f1000research.13923.1 (PMC5941245; doi:10.12688/f1000research.13923.1)
Supplement: Supplementary file 2 [file f1000research-7-15136-s0001.tgz › 1198a7eb-0a05-46e0-a2f5-a643503361b5.docx]

**Switching from biosimilar (Basalin) to originator (Lantus) insulin glargine is effective in Chinese patients with diabetes mellitus: a retrospective study**

**Supplementary materials**

Supplementary Table 1. Summary of insulin doses during initial treatment with Basalin insulin glargine and after switching to Lantus for patients who received basal-bolus therapy only without OADs

| Insulin dose, IU/kg/day | Basalin insulin glargine | | Insulin switch | Lantus insulin glargine | | P-value^a^ (Basalin final dose vs. originator final dose) |
| --- | --- | --- | --- | --- | --- | --- |
|  | (mean duration 11.4 [6.93] days) | |  | (mean duration 6.4 [7.38] days) | |  |
|  | Initial dose | Final dose |  | Initial dose | Final dose |  |
| Insulin glargine | 0.2 (0.07) | 0.24 (0.08) |  | 0.25 (0.09) | 0.25 (0.09) | 0.1321 |
| P-value, initial vs. final dose^a^ |  | 0.0001 |  |  | 0.6719 |  |
| Prandial insulin^b^ | 0.32 (0.08) | 0.4 (0.15) |  | 0.41 (0.15) | 0.4 (0.16) | 0.8429 |
| P-value, initial vs. final dose^a^ |  | <.0001 |  |  | 0.483 |  |

 Supplementary Table 2. Seven-point blood glucose summary for patients who switched due to suboptimal glycemic control as recorded in medical records

|  | Study time point (n=22) | | | | P-value^b^ | | |
| --- | --- | --- | --- | --- | --- | --- | --- |
| Blood glucose, mmol/L^a^ | Hospital admission | Basalin initiation | Insulin  switch | Hospital discharge | Basalin initiation vs. hospital admission | Insulin switch vs. Basalin initiation | Hospital discharge vs.  insulin switch |
| FBG | 10.89 (3.91) | 10.17 (3.59) | 8.51 (2.18) | 7.37 (1.7) | 0.1210 | 0.0540 | 0.0337 |
| Post-breakfast BG | 12.77 (3.7) | 12.28 (3.34) | 11.03 (3.39) | 9.4 (2.46) | 0.4004 | 0.2561 | 0.0607 |
| Pre-lunch BG | 10.92 (3.81) | 10.55 (2.93) | 9.19 (2.65) | 7.42 (1.9) | 0.6695 | 0.0237 | 0.0068 |
| Post-lunch BG | 12.22 (4.53) | 11.49 (4.45) | 10.03 (2.94) | 9.98 (2.91) | 0.1305 | 0.1954 | 0.9352 |
| Pre-dinner BG | 9.63 (3.29) | 9.46 (2.86) | 8.91 (3.29) | 8.67 (3.15) | 0.7423 | 0.2621 | 0.7033 |
| Post-dinner BG | 11.5 (3.72) | 11.52 (2.85) | 10.08 (3.09) | 9 (2.49) | 0.9702 | 0.0598 | 0.1530 |
| Pre-bedtime BG | 11.31 (4.44) | 11.38 (4.31) | 9.36 (3.42) | 8.12 (1.87) | 0.9283 | 0.0434 | 0.1131 |

Supplementary Table 3. Summary of insulin doses during initial treatment with Basalin insulin glargine and after switching to Lantus for patients who switched to Lantus due to suboptimal glycemic control as recorded in medical records

| Insulin dose, IU/kg/day | Basalin insulin glargine | | Insulin switch | Lantus insulin glargine | | P-value^a^ (Basalin final dose vs. originator final dose) |
| --- | --- | --- | --- | --- | --- | --- |
|  | (mean duration 8.4 [5.92] days) | |  | (mean duration 11.7 [10.33] days) | |  |
|  | Initial dose | Final dose |  | Initial dose | Final dose |  |
| Insulin glargine | 0.21 (0.09) | 0.25 (0.08) |  | 0.27 (0.1) | 0.26 (0.1) | 0.5069 |
| P-value, initial vs. final dose^a^ |  | 0.0485 |  |  | 0.7562 |  |
| Prandial insulin^b^ | 0.33 (0.08) | 0.43 (0.13) |  | 0.44 (0.13) | 0.41 (0.14) | 0.5085 |
| P-value, initial vs. final dose^a^ |  | 0.0021 |  |  | 0.4096 |  |

Supplementary Figure 1. Seven-point blood glucose measurement for Chinese patients with diabetes mellitus at hospital admission, Basalin insulin glargine initiation, insulin switch, and hospital discharge


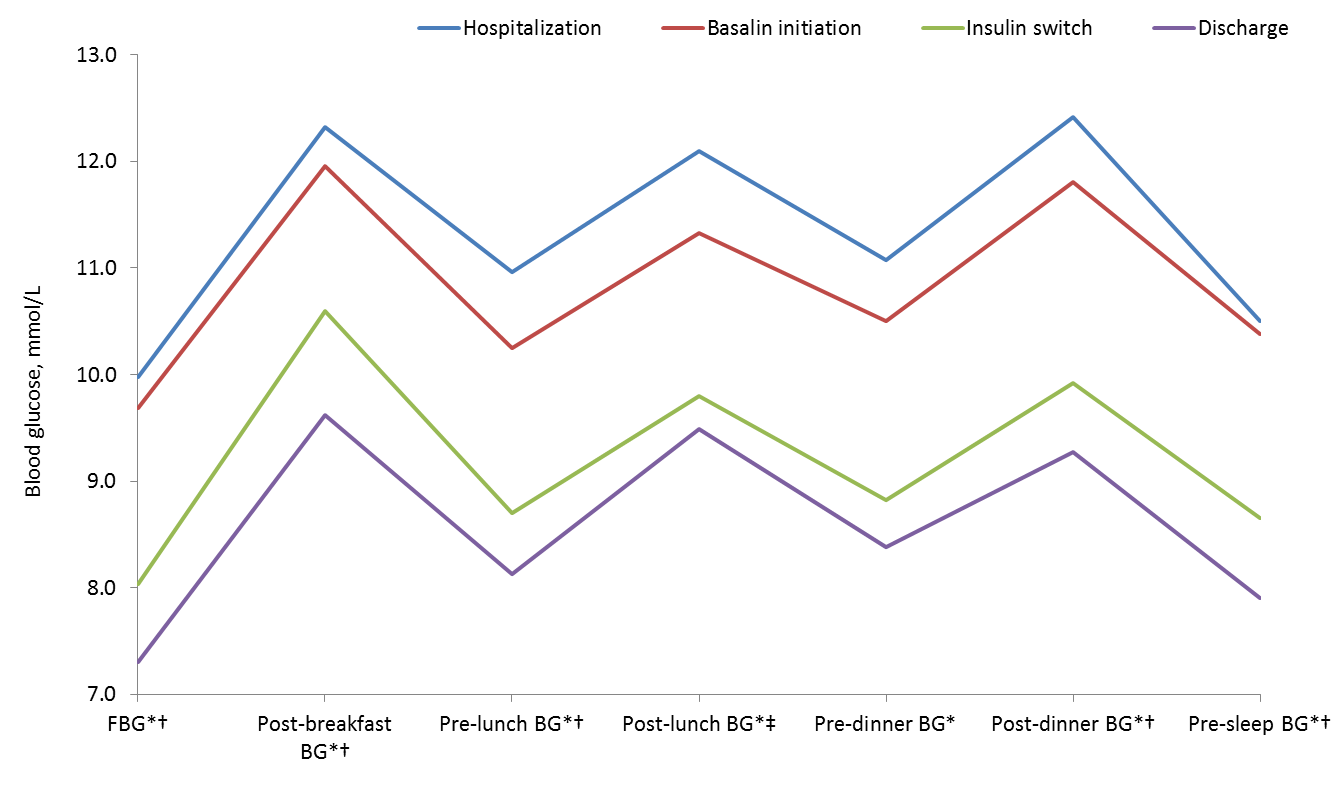


*p<0.05 for insulin switch vs. treatment initiation.

†p<0.05 for hospital discharge vs. insulin switch.

‡p<0.05 for Basalin initiation vs. hospitalization.

Supplementary Figure 2. Seven-point blood glucose measurement for Chinese patients with diabetes mellitus who received basal-bolus treatment only with no OAD use (n=62) at hospital admission, Basalin insulin glargine initiation, insulin switch, and hospital discharge


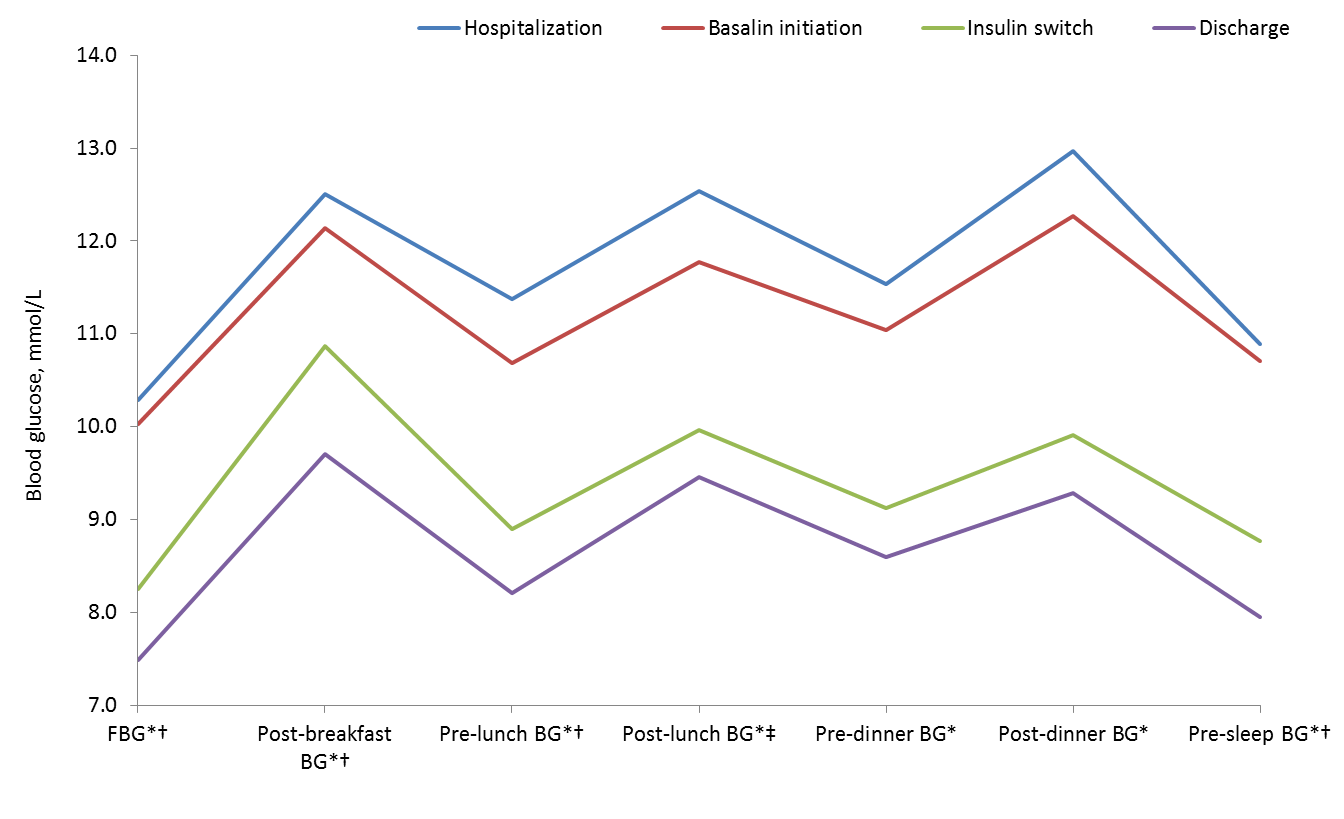


*p<0.05 for insulin switch vs. treatment initiation.

†p<0.05 for hospital discharge vs. insulin switch.

‡p<0.05 for Basalin initiation vs. hospitalization.
