# Supplementary material for: Switching from biosimilar (Basalin) to originator (Lantus) insulin glargine is effective in Chinese patients with diabetes mellitus: a retrospective chart review
Source: F1000Res. 2018 Apr 18;7:477. [Version 1] doi: 10.12688/f1000research.13923.1 (PMC5941245; doi:10.12688/f1000research.13923.1)
Supplement: Peer review history

# Peer review: Referee response for version 1

**DOI:** 10.5256/f1000research.15136.r33299

**Published:** 2018-05-11

**Authors:** Xia Li

**Affiliations:** 1 Department of Metabolism and Endocrinology, The Second Xiangya Hospital, Central South University, Changsha, China

I recommend the indexing of this paper in your journal based on the following considerations:

 1.   This is one of the few papers discussing the effectiveness and safety between biosimilar insulin and originator insulin in real world clinical practice in a Chinese diabetes population. It releases the message that biosimilar is potentially different in effectiveness from originator as reported by previous papers. It helps provide a clinical implication to physicians and raise the necessity of long term observation or well designed trials to identify the differences in real world at the same time.

 2.   Despite the limitation of small sample size, short term treatment duration and potential bias mentioned in this paper, the authors smartly designed two subgroup analysis. It also showed that the mean FBG was lower after switching from Basalin to Lantus , 8.25mM vs. 7.49mM(p=0.0217,for purely basal-bolus regimen with no OAD population) and 8.51mM vs.7.37mM(p=0.0337,for recorded suboptimal glycemic control population). The conclusion is consistent as that in the whole group.

 3.   The graphs should be displayed in a simple way to be well understood.

I have read this submission. I believe that I have an appropriate level of expertise to confirm that it is of an acceptable scientific standard.

# Peer review: Referee response for version 1

**DOI:** 10.5256/f1000research.15136.r33296

**Published:** 2018-05-08

**Authors:** Yan Li

**Affiliations:** 1 Department of Endocrinology, Sun Yat-Sen Memorial Hospital, Sun Yat-Sen University, Guangzhou, China

1. The authors explored 73 diabetes patients treated with basal insulin regimen, starting from Basalin and then switched to Lantus in hospitalized setting based on electronic medical records from 2005 to 2016. The main outcomes showed further fasting blood  glucose reduction with similar insulin required and similar safety profile. Meanwhile, the study released the same message that basal insulin was not fully titrated as other studies in Chinese diabetes patient, only about 0.05U/kg insulin dose increased (0.19U/kg to 0.24U/kg) at discharge compared with prandial insulin (0.18U/kg insulin dose increased from 0.32 to 0.40 IU/kg) after almost  three weeks of hospitalization. The titration situation was supposed to be related to the limited clinical experience of the two basal insulin, Lantus and Basalin, which was launched in 2004, 2005 respectively and data included for analysis between 2005 and 2016, with diversified practice. But the outcomes did reflect the effectiveness of Lantus and Basalin in real life.
2. In addition to the limitation mentioned in this article, some limitation should also be considered. First, the treatment duration of Lantus was 6.6(7.36),which meant the minimum treatment duration might be about 1-2 day. Therefore, it is much more appropriate to explore the data in steady state,for example 4-5 day after the first dosing. Second, the data was from a single site and the patient number was small for a retrospective study to address a strong conclusion but authors has realized it and provided a quite fair conclusion on the effectiveness.
3. The article was well written and I personally agree it can be considered for publication in your journal.

I have read this submission. I believe that I have an appropriate level of expertise to confirm that it is of an acceptable scientific standard.

# Peer review: Referee response for version 1

**DOI:** 10.5256/f1000research.15136.r33298

**Published:** 2018-05-04

**Authors:** Benli Su

**Affiliations:** 1 Department of Endocrinology, The Second Affiliated Hospital of Dalian Medical University, Dalian, China

As a peakless long-action insulin analogue, Insulin glargine has been widely used in China, as basal insulin initiating paradigm become more and more accepted. Biosimilar insulin glargine has been on market for many years. As debate if biosimilar is as effective as originator continued, we also once noticed three patients switching from biosimilar glargine to originator resulted in improved glycemic control without increase in dosage. In the report the authors retrieved data of inpatient electronic record, studied retrospectively if the switching from biosimilar insulin glargine to its originator would improve patients glycemic control. They found it did improve patient glucose control after the switching. The authors discussed the possible reasons. But as reported, this is a retrospectively study, there was no control. From the authors schematic description of data retrieval, we noticed there still had around 20 patients who switched their insulin from originator to biosimilar, although the number was small, this might still be used as a control. We discussed our case report one possible reason of improvement in glucose control might be due to inpatient diabetes education, strict dietary restriction. The adding of these “control” patients  would provide more strong evidence supporting the use of originator.

I have read this submission. I believe that I have an appropriate level of expertise to confirm that it is of an acceptable scientific standard, however I have significant reservations, as outlined above.

# Peer review: Referee response for version 1

**DOI:** 10.5256/f1000research.15136.r33295

**Published:** 2018-05-03

**Authors:** Longyi Zeng

**Affiliations:** 1 The Third Affiliation Hospital, Sun Yat-Sen University, Guangzhou, China

1. This study is well structured with a clear objective to explore a very common scientific question in daily clinical practice. In this study, authors reported a significant FBG reduction(additional -0.67mmol/L)  and  better overall hyperglycemia control after switching from Basalin to Lantus at similar insulin dosage in hospitalized diabetes patients, mostly are type 2 diabetes patients. This outcome helps provide an initial answer on the efficacy and safety difference between biosimilar insulin and originator insulin in real clinical practice for physicians. This study also implies  the importance of FBG control on overall glycemic control for physicians.
2. As a retrospective chart study, the outcomes inevitably limited by some confounding factors as discussed in the paper.  To better verify the outcome, authors explored subgroup analysis in basal bolus regimen and recorded suboptimal glycemic control population, from which with similar outcomes as in total population. Sufficient and valid data in Lantus switching to Basalin as control group or crossover design study may provide further useful and robust information on efficacy, safety evaluation.
3. After a careful review of this manuscript, I do think it appropriate for publication in F1000 Research.

I have read this submission. I believe that I have an appropriate level of expertise to confirm that it is of an acceptable scientific standard.
